# Iron homeostasis landscape informs patient survival and neoadjuvant chemotherapy response in cancer

**DOI:** 10.1186/s13040-026-00589-3

**Published:** 2026-07-24

**Authors:** Xiaoqing Fan, Kangna Cao, Xu Steven Xu, Xiaoyu Yan

**Affiliations:** 1 School of Pharmacy, Faculty of Medicine, The Chinese University of Hong Kong, Hong Kong SAR, P. R. China; 2 Guangdong-Hong Kong-Macao Joint Laboratory for New Drug Screening, School of Pharmacy, The Chinese University of Hong Kong, Hong Kong SAR, P. R. China; 3 Clinical Pharmacology and Quantitative Science, Genmab Inc., Princeton, NJ USA; 4 The Chinese University of Hong Kong, 8th Floor, Lo Kwee-Seong Integrated Biomedical Sciences Building, Area 39, Shatin, N.T. Hong Kong, China

**Keywords:** Iron homeostasis, Iron risk score, Time-to-event, Survival, Chemotherapy response

## Abstract

**Background:**

Cancer cells frequently alter iron metabolism by increasing uptake or decreasing export, promoting oncogenesis and proliferation. However, the quantitative relationship between iron homeostasis and cancer remains underexplored.

**Methods and results:**

We simplified these alterations into measurable changes in the expression levels of iron transport-related genes. We investigated the expression of iron transport-related genes by comparing genomic data of 478 human cancer cell lines to 105 normal tissue samples, as well as analyzing clinically paired 6903 tumor to 709 normal samples. We revealed that cancer cells exhibited increased expression of transferrin receptor 1 (TFR1) or CD44 for iron uptake and decreased expression of ferroportin for iron efflux to favor iron accumulation. Using machine learning, we identified TFR1, CD44, and ferroportin as the top three features consistently associated most with patient survival. We developed a cancer-specific iron risk score (CIRS) using parametric time-to-event models based on RNA-seq data from TFR1, CD44, and ferroportin in 4723 patients spanning 15 cancers. The CIRS, cross-validated in five external datasets, significantly stratified survival risk for various cancers, and predicted chemotherapy response in two independent breast cancer pharmacogenomic datasets.

**Conclusions:**

Our study enhances the understanding of the relationship between iron homeostasis, survival, and chemotherapy response, laying the groundwork for iron-targeted therapies.

**Supplementary Information:**

The online version contains supplementary material available at https://doi.org/10.1186/s13040-026-00589-3.

## Background

Iron is a crucial component required for a variety of vital physiological processes, such as DNA synthesis and energy metabolism that is indispensable to cell growth and division [1]. The regulation of cellular iron metabolism is mainly composed of iron uptake and efflux that work together to tightly maintain intracellular iron homeostasis. Emerging evidence has demonstrated that altered iron homeostasis is a hallmark observed in numerous types of tumors. In cancer cells, enhanced iron uptake and/or decreased iron efflux is essential to increase the intracellular iron amount to support their increased demands of survival, rapid cell division, and metastasis [2].

Previous studies have offered preliminary insights into the role of multiple dysregulated iron-related genes on patient survival [3, 4]. However, these efforts analyzed the genes separately and neglected the exploration of the full spectrum of iron homeostasis alterations in cancer progression [3–5]. The amount of iron required by diverse tumors is different due to their remarkable heterogeneity. How the expression level of genes that control iron uptake and efflux changes and coordinates to generate a unified effect on iron homeostasis is an important open question. We still have limited knowledge of the association between the expression level of iron-transport genes that are responsible for maintaining iron homeostasis and cancer patients’ survival across various cancers. These quantitative interconnections are important for guiding novel interventions for cancer therapy that involve iron homeostasis perturbations, such as iron-chelator-based therapy and ferroptosis-inducing drugs [6–8]. Moreover, investigating the biological relevance and clinical significance of iron homeostasis in larger patient cohorts to stratify high-risk individuals at an early clinical stage holds promise to facilitate precision oncology, turning the unique iron metabolic properties of cancer cells to therapeutic advantage [7, 9, 10].

**Fig. 1:**
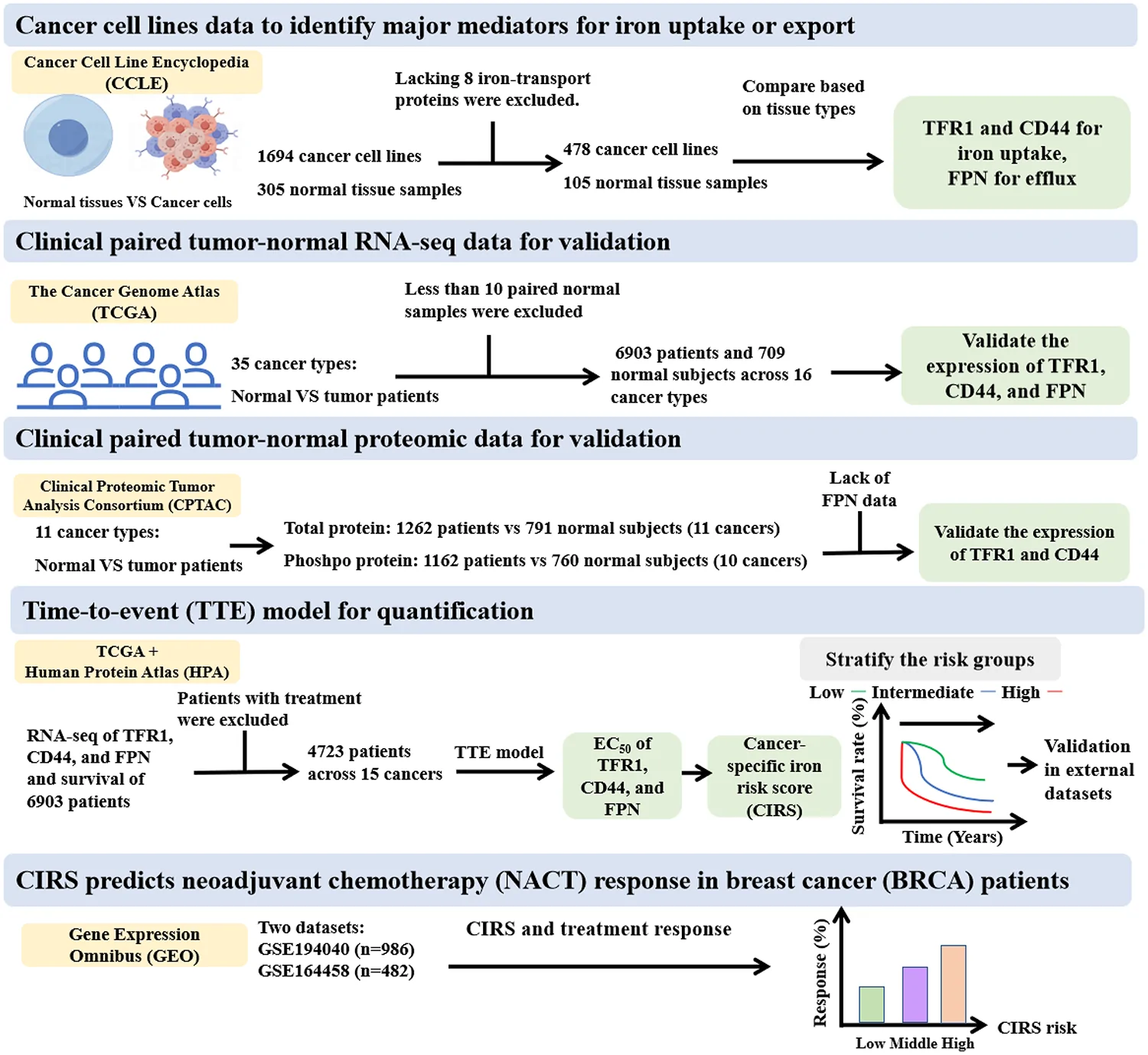
Study overview. First, we extracted data from Expression Atlas (https://www.ebi.ac.uk/gxa/home) to compare the expression level of eight iron-transport proteins that are involved in iron uptake and efflux between normal tissue samples and cancer cells. A total of 1524 human cancer cell lines and 105 human normal tissue samples were included, which are classified into 24 tissues. Next, we further compared the RNA and protein expression levels of three transporters (TFR1, CD44, and FPN) between cancer patients and normal subjects using the clinical data from The Cancer Genome Atlas (TCGA) database and Clinical Proteomic Tumor Analysis Consortium (CPTAC) database. Then, we developed a parametric time-to-event model using individual RNA expression data of TFR1, CD44, and FPN, and survival data from TCGA and Human Protein Atlas (HPA) database to generate cancer-specific risk score (CIRS) for 15 types of cancers. Subsequent survival analysis and external validation were performed using CIRS to stratify the risk groups in different types of cancer. The CIRS was used to predict neoadjuvant chemotherapy response in breast cancer (BRCA) patients using two independent BRCA pharmacogenomic datasets

In this study, we comprehensively compared the expression level of eight iron-transport genes in 478 cancer cell lines with corresponding 105 normal human tissue samples (Fig. 1). We further validated the findings in cancer cell lines by comparing clinically paired 6903 tumor to 709 normal tissue samples. Utilizing machine learning, we comprehensively explored correlations between these transporters and the survival of cancer patients, assessing the magnitude of their contributions to survival prediction. Leveraging population longitudinal and parametric time-to-event (TTE) modeling strategies [11], we developed TTE models using the expression profile of the three major transporters that control iron uptake and efflux in 4723 cancer patients and their survival outcomes, which quantified the contribution of the expression level of these three transporters on the outcome of patients across fifteen types of cancers. We created and validated a cancer-specific iron risk score (CIRS) based on TTE modeling results, which allowed us to make a quantitative inference from early continuous RNA-seq data to predict the long-term individual survival outcomes of patients for the first time in common cancer types. Notably, CIRS can also predict neoadjuvant chemotherapy (NACT) response in breast cancer (BRCA) patients from 2 independent BRCA pharmacogenomic datasets.

## Methods

### Cancer cell lines genomic data to identify major mediators for iron uptake or export

We searched the original literature for iron-transport genes (including uptake and efflux) that are responsible for maintaining iron homeostasis and reviews of iron metabolism to exclude unrelated genes and add recently reported genes. Finally, eight iron-transport genes, including seven iron uptake genes ransferrin receptor 1 (TFR1, encoded by TFRC), cluster determinant 44 (CD44, encoded by CD44), divalent metal transporter 1 (DMT1, encoded by SLC11A2), heme carrier protein 1 (HCP1, encoded by SLC46A1), ion channels Zrt-/Irt-like protein 8 (ZIP8, encoded by SLC39A8) and ZIP14 (encoded by SLC39A14), lipocalin-2 (LCN2, encoded by SLC22A17), and one only known iron efflux gene ferroportin (FPN, encoded by SLC40A1), were included in this study (Table S1) [12]. The expression of the eight iron-transport genes across 1694 cancer cell lines from Cancer Cell Line Encyclopedia (CCLE) [13], and 305 human normal tissue samples were downloaded from Expression Atlas (https://www.ebi.ac.uk/gxa/home) [14]. The Fragments Per Kilobase of transcript per Million (FPKM) mapped reads values were utilized for qualitative comparisons of gene expression levels. The RNA-seq results that lack any one of the 8 iron-transport genes are regarded as incomplete and were excluded (Fig. 1). Finally, 478 cancer cell lines and 105 normal tissue samples were included (Table S2). We divided the samples into 24 different tissue types, and compared the expression levels of the eight iron-transport genes between cancer cell lines and normal tissues using multiple unpaired *t* test in GraphPad Prism (version 9.5.1), with *p* < 0.05 considered to indicate statistically significant differences.

### Clinical paired tumor-normal RNA-seq and proteomic data comparison

To screen and validate the major mediators for iron uptake or export in cancer patients, we collected human data from UALCAN (https://ualcan.path.uab.edu/index.html), a comprehensive database that integrates publicly available cancer OMICS data (Table S3, including The Cancer Genome Atlas [TCGA] and Clinical Proteomic Tumor Analysis Consortium [CPTAC] database) [15]. The RNA-seq data of 3 iron transport-related genes in cancer patients, including TFR1, CD44, and FPN, were compared with the corresponding data from normal subjects in the TCGA, using Transcripts Per Million (TPM) as the expression values to ensure consistency and comparability across datasets. We only included cancer types that contained more than 10 paired normal samples to ensure the strength of statistical power (Fig. 1). Therefore, 6903 TCGA patient samples and 709 normal subject samples across 16 cancer types, including bladder urothelial carcinoma (BLCA), breast invasive carcinoma (BRCA), colon adenocarcinoma (COAD), esophageal carcinoma (ESCA), head and neck squamous cell carcinoma (HNSC), kidney chromophobe (KICH), kidney renal clear cell carcinoma (KIRC), kidney renal papillary cell carcinoma (KIRP), liver hepatocellular carcinoma (LIHC), lung adenocarcinoma (LUAD), lung squamous cell carcinoma (LUSC), prostate adenocarcinoma (PRAD), rectum adenocarcinoma (READ), stomach adenocarcinoma (STAD), thyroid carcinoma (THCA), and uterine corpus endometrial carcinoma (UCEC), were analyzed.

The protein expression data from the CPTAC database, including total protein and phoshpo protein of the corresponding iron transport-related genes, was further analyzed and compared with the corresponding normal subjects based on the tissue types. Since the FPN data is not available in the database, only TFR1 and CD44 data were analyzed. The protein expression levels are logarithmically transformed and z-score normalized for patients and normal subjects, ensuring that the data are uniformly scaled and suitable for cross-sample comparisons. Dataset preparation and statistical evaluation were performed in the R program (version 4.2.0, www.r-project.org).

### Machine learning (ML) analysis for gene importance ranking

Next, to further sort the importance of the eight iron-transport genes to the survival prediction, we constructed a ML model by using the Extreme Gradient Boosting (XGBoost). We fed the eight genes after Bonferroni corrections derived from Cox proportional hazard regressions into the XGBoost model, ranking each gene based on its contribution to the predictive performance of the model [16], with TPM values used as the expression metric. The performance was judged by the information gain, which can be considered as the gene’s capability to predict patients’ survival. The ML analysis was performed by R v.4.2.0 using the xgboost (version 1.7.8.1) and caret (version 6.0–94) packages.

### Parametric time-to-event (TTE) models

Based on the feature screening and XGBoost ranking results, we developed and evaluated parametric TTE models to evaluate the impact of patient iron transport-related genes (TFR1, CD44, and FPN) expression level on the observed overall survival (OS), and to stratify the iron homeostasis-based risk. RNA-seq data were used because they are more complete than the protein expression data (Fig. 1) [11]. The RNA-seq data of individual patients, using TPM values as the expression metric, along with their corresponding demographics and characteristics, were collected from the Human Protein Atlas (HPA) database [17] and the National Cancer Institute’s Genomic Data Commons data portal (https://portal.gdc.cancer.gov/v1/) (Table S4).

Different baseline hazard models (exponential, Weibull, and Gompertz) were tested in different cancers, and the Gompertz models (Eq. 1) were selected to describe the OS for BLCA, BRCA, COAD, KIRC, and STAD, while the Weibull models (Eq. 2) were used to describe the OS for HNSC, KICH, KIRP, LIHC, LUAD, LUSC, PRAD, READ, THCA, and UCEC, based on the decrease in the objective function value and simulation-based visual predictive checks (VPCs), a diagnostic tool for evaluating the performance of the model [18]. The impact of baseline continuous covariates TFR1, CD44, and FPN was incorporated into the parametric TTE models and was evaluated using sigmoid functions.

1 $$\eqalign{ h\left( t \right) & = {\lambda _{OS}} \cdot {e^{\alpha \cdot t}} \cdot \left( {1 + {{{E_{max}}_{TFR1} \cdot TFR{1^{\gamma 1}}} \over {TFR{1_{50}}^{\gamma 1} + TFR{1^{\gamma 1}}}}} \right) \cr & \cdot \left( {1 + {{{E_{max}}_{CD44} \cdot CD{{44}^{\gamma 2}}} \over {CD{{44}_{50}}^{\gamma 2} + CD{{44}^{\gamma 2}}}}} \right) \cdot \left( {1 + {{{E_{max}}_{FPN} \cdot FP{N^{\gamma 3}}} \over {FP{N_{50}}^{\gamma 3} + FP{N^{\gamma 3}}}}} \right) \cr}$$

2 $$\eqalign{ h\left( t \right) & = {\lambda _{OS}} \cdot \alpha \cdot {\left( {{\lambda _{OS}} \cdot t} \right)^{\alpha - 1}} \cdot \left( {1 + {{{E_{max}}_{TFR1} \cdot TFR{1_t}^{\gamma 1}} \over {TFR{1_{50}}^{\gamma 1} + TFR{1_t}^{\gamma 1}}}} \right) \cr & \cdot \left( {1 + {{{E_{max}}_{CD44} \cdot CD{{44}_t}^{\gamma 2}} \over {CD{{44}_{50}}^{\gamma 2} + CD{{44}_t}^{\gamma 2}}}} \right) \cdot \left( {1 + {{{E_{max}}_{FPN} \cdot FP{N_t}^{\gamma 3}} \over {FP{N_{50}}^{\gamma 3} + FP{N_t}^{\gamma 3}}}} \right) \cr}$$

Where h(t) is the hazard of death, $\:{\lambda\:}_{OS}$ is the hazard scale factor, α is the hazard shape factor, $\:{{E}_{max}}_{TFR1}$, $\:{{E}_{max}}_{CD44}\:$, and $\:{{E}_{max}}_{FPN}$ are maximum effect parameters of TFR1, CD44, and FPN on OS, respectively. $\:{TFR1}_{50}$, $\:{CD44}_{50}$, and $\:{FPN}_{50}$ are half-expression levels of TFR1, CD44, and FPN that are needed to reach the maximum effect on OS, respectively. γ1, γ2, and γ3, are the slope-factor for the effect of TFR1, CD44, and FPN, respectively.

Only patients with baseline TFR1, CD44, and FPN expression level data were included to compare the impact of iron homeostasis on OS. The patients who received treatment or therapy were excluded to minimize the influence of post-study medication.

Using the final covariate model and the distribution of the TCGA datasets, simulation-based (1000 replicates) VPCs were compared for different models [19]. For each simulation, Kaplan–Meier proportions of the surviving patients were computed across time for evaluation of median OS and the 90% confidence intervals around simulations were included based on the 5th and 95th percentiles of the estimated covariate effect parameters.

The parametric TTE analysis was performed using the first-order conditional estimation method with interaction algorithm in NONMEM (Version 7.5, Icon Development Solutions, Ellicott City, MD, USA). The use of NONMEM was facilitated by Perl-speaks-NONMEM (version 4.9.6, http://psn.sourceforge.net/docs.php). The ordinary differential equations were solved by the ADVAN6 subroutine.

### Iron homeostasis-based risk stratification

Based on the TTE modeling estimates, we developed a CIRS to leverage baseline iron homeostasis profiles for predicting long-term survival outcomes across multiple cancer types. We divided the patients into tertile groups based on stratified CIRS. Specifically, the risk of a cancer patient was deemed to be substantially associated with the expression level of TFR1, CD44, and FPN and was classified as (Table S5): (i) For TFR1 and CD44 expression levels higher than $\:{TFR1}_{50}$ and $\:{CD44}_{50}$ values and the FPN expression level lower than $\:{FPN}_{50}$ values, the patient is regarded as high risk because this profile favors iron accumulation via increased iron uptake and decreased iron efflux; (ii) For TFR1 and CD44 expression levels lower than $\:{TFR1}_{50}$ and $\:{CD44}_{50}$ values, while the FPN expression level higher than $\:{FPN}_{50}$ values, the patient is regarded as low risk because this profile reduces iron accumulation via decreased iron uptake and increased iron efflux; (iii) The patient outside these two groups is classified into the intermediate risk. Then, the relationship between CIRS and patient survival was plotted with the Kaplan–Meier survival curves using the Survival (version 3.5.8) and Survminer (version 0.4.9) R packages.

### External cross-validation of the stratification results

For external cross-validation of our method, five RNA-seq datasets with clinical information were included [20–24], including *n* = 708 patients with Multiple Myeloma from the CoMMpass study (NCT01454297), *n* = 657 patients with Gliomas from the Chinese Glioma Genome Atlas (CGGA), *n* = 72 patients with high-grade serous carcinomas (HGSCs), *n* = 20 patients with lung cancer from the Sherlock-Lung study, and *n* = 45 patients with small cell lung cancer. The gene expression values were kept consistent with the five original RNA-seq datasets. The expression units did not affect the model’s performance, as the model calculated the corresponding values for TFR1_50_, CD44_50_, and FPN_50_ based on the respective gene expression values. Subsequently, it is only necessary to ensure consistency with these corresponding gene expression values to apply this CIRS. The data accession numbers and references for these datasets and detailed clinical information are provided in Supplementary Table S6.

### Prediction of neoadjuvant chemotherapy (NACT) response in patients

Iron is a crucial component for DNA and RNA synthesis [1]. Therefore, we hypothesized that cancer cells that have more iron content may be more sensitive to the DNA damage chemotherapeutical drugs. To gain insights into the NACT-induced effects on evolutionary trajectories of iron homeostasis, we firstly analyzed iron homeostasis RNA-seq profiling of a longitudinal BRCA cohort of 146 patients including 110 pairs of serial tumor biopsies collected before treatment (T1), three weeks after the first cycle of NACT (T2), and at the time of surgery (T3) for patients who did not achieve pCR. The dataset GSE123845 (Clinical Trial ID: NCT02591966) was downloaded from Gene Expression Omnibus (GEO, https://www.ncbi.nlm.nih.gov/geo/query/acc.cgiacc=GSE123845), using TPM values as the expression metric [25]. Specifically, there are 44 patients consisting of 21 pCR cases that have paired T1 and T2 samples, and 23 residual disease (RD) cases that have paired T1, T2, and T3 samples.

We further took BRCA as an example to determine whether iron homeostasis alterations could predict the NACT response in cancer patients, because there are two large independent datasets (GSE194040 and GSE164458) from GEO including treatment response and RNA-seq data of TFR1, CD44, and FPN (Table S7), with TPM values used as the expression metric [26, 27]. Whole transcriptome RNA-seq was performed on pre-treatment research biopsies. Patients in the two datasets (*n* = 986 in GSE194040 and *n* = 482 in GSE164458) were stratified into three subgroups (low, middle, and high risk) based on the predefined CIRS. The response rate was calculated as the number of patients that achieved pathologic complete response (pCR) divided total number of patients in the subgroup, and the response rate among the three groups was compared using the Chi-Square test in SPSS (version 22.0).

## Results

### The pan-cancer expression landscape of the iron transport‐related genes

The study overview is shown in Fig. 1. Because the intracellular iron amount in cancer cells is increased, we hypothesized that this is achieved by either the increased expression of iron uptake genes, decreased expression of iron efflux gene, or both. Based on the literature review, iron uptake is mainly organized by TFR1. This well-studied pathway involves the binding of di-ferric transferrin (TF) to TFR1, endocytosis of the TFR1/TF-Fe complex, and subsequent exit of iron from the endosome to the cytoplasm via DMT1 [12]. In addition, LCN2, CD44, and non–transferrin-bound iron (NTBI) that is transported directly into the cytoplasm via HCP1, transmembrane ion channels ZIP8 and ZIP14, all have been reported to be involved in iron uptake to fuel the labile iron pool (LIP) [28–31]. The iron in the LIP can be utilized to synthesize iron-containing proteins, while excess iron can be exported outside the cell mainly through FPN. We therefore compare the alterations of these 8 genes across cancers, including 7 genes for iron uptake and 1 gene for iron efflux (Table S1).

We obtained RNA-seq data of the 8 genes in 478 cancer cell lines from the CCLE database and 105 human normal tissue samples from Expression Atlas (Table S2). We divided the samples into 24 different tissue types, and compared the expression levels of the eight iron-transport genes between cancer cell lines and normal tissues. We found that, across tissue types, at least 1 iron uptake gene, either TFR1 or CD44, overexpressed, or the iron efflux gene FPN was decreased, or both, in the analysis of cancer cell lines compared to the corresponding normal tissue samples (Figure S1). Notably, for the genes that were reported to participate in iron uptake, including DMT1, HCP1, LCN2, ZIP8, and ZIP14, their expression levels were not significantly increased in most cancers, and thus were excluded in the subsequent studies (Figure S2 and Figure S3).

**Fig. 2:**
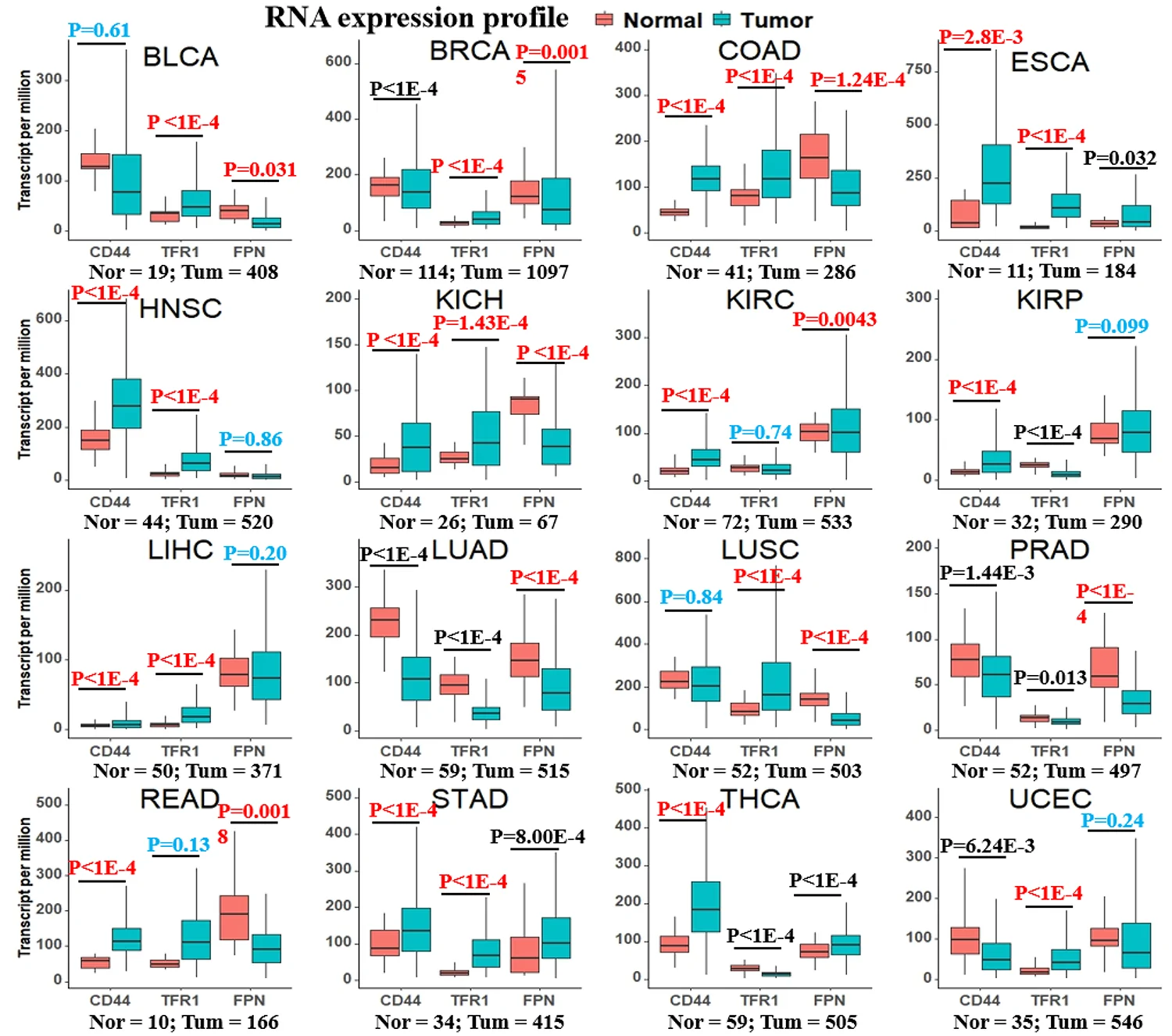
Cancer patients demonstrate higher expression of transferrin receptor (TFR1) or CD44 for iron uptake, or lower expression of SLC40A1 (ferroportin, FPN) for iron export, or both, that favor iron accumulation to promote oncogenesis and cell proliferation. RNA level of TFR1, CD44, and SLC40A1 in normal subjects and the corresponding cancer patients based on cancer types from the TCGA database. p value in black color means the expression level trend of the protein is different from our hypothesis, while the p value in red color means the expression level trend of the protein is consistent with our hypothesis. P value in blue color means no significant difference. Cancer types are labeled using the TCGA study abbreviations. BLCA: Bladder urothelial carcinoma; BRCA: Breast invasive carcinoma; COAD: Colon adenocarcinoma; ESCA: Esophageal carcinoma; HNSC: Head and Neck squamous cell carcinoma; KICH: Kidney chromophobe; KIRC: Kidney renal clear cell carcinoma; KIRP: Kidney renal papillary cell carcinoma; LIHC: Liver hepatocellular carcinoma; LUAD: Lung adenocarcinoma; LUSC: Lung squamous cell carcinoma; PRAD: Prostate adenocarcinoma; READ: Rectum adenocarcinoma; STAD: Stomach adenocarcinomna; THCA: Thyroid carcinoma; UCEC: Uterine corpus endometrial carcinoma

We validated this result in humans using RNA-seq data of the three genes from paired tumor-normal samples in TCGA. The results across 16 types of cancers are consistent with cancer cell line analysis and further confirm our hypothesis (Fig. 2).

To investigate whether the principle is applicable across different cancers at the protein level, we evaluated the expression level changes of these three genes using the proteomic data from the independent CPTAC datasets. However, only TFR1 and CD44 data are available. As shown in Figure S4, despite that total protein expression levels of both TFR1 and CD44 are not increased significantly in BRCA, LUAD, and ovarian cancer, the expression levels of TFR1 and CD44 in other 8 distinct cancer types support the RNA-seq results. Moreover, the phosphor-protein expression of TFR1 and CD44 suggests that at least 1 iron uptake protein, either TFR1 or CD44, should increase across the analyzed 10 types of cancers (Figure S5).

Altogether, both the in vitro cancer cell lines analysis and the human validation analyses using RNA-seq and proteomics data confirmed that, to favor iron accumulation to promote oncogenesis and cell proliferation in cancer cells, at least 1 iron uptake protein, either TFR1 or CD44, was overexpressed, or the iron efflux protein FPN was decreased, or simultaneously.

### Machine learning identified key genes associated with cancer patients’ overall survival (OS)

Of the eight iron transport genes, we further ranked their importance to the prediction of cancer patients’ OS by applying ML approach. The RNA-seq data of these genes from the HPA database was used. A total of 15 cancers within the compared 16 cancers had individual long-term survival and RNA-seq data, including BLCA, BRCA, COAD, HNSC, KICH, KIRC, KIRP, LIHC, LUAD, LUSC, PRAD, READ, STAD, THCA, and UCEC. The patient characteristics are summarized in Table S4.

Notably, we found that the three genes, TFR1, CD44, and FPN, consistently ranked top three in predicting OS (Figure S6). In detail, TFR1 was the strongest predictor of BLCA, KICH, LUAD, LUSC, and PRAD. CD44 was the strongest predictor of BRCA, COAD, KIRC, KIRP, LIHC, READ, STAD, THCA, and UCEC. FPN was the strongest predictor of HNSC. Despite only two genes (TFR1 and FPN) showing significance with OS due to limited sample size in KICH (*n* = 67), TFR1, CD44, and FPN maintained the most robust associations with OS spanning the fifteen cancer types. Therefore, we ultimately chose the three genes, TFR1, CD44, and FPN for subsequent analyses.

### Iron homeostasis-based TTE models predict long-term survival in patients across diverse cancers

To investigate the influence of the expression level of TFR1, CD44, and FPN on patients’ OS across cancers, we developed parametric TTE models using the RNA-seq data of these three genes.

Despite the heterogeneous contribution of the three genes to OS, an increase in TFR1 and CD44 raised the hazards of death and disease progression, whereas higher FPN was beneficial for survival. The magnitude of baseline hazards was quite different across the 15 cancers, in line with the observed event times (Table 1).

**Table 1:** Final parameters from time-to-event analysis of overall survival in patients across 15 cancers from TCGA with baseline measurements of TFR1, CD44, and FPN

Cancer types	Model Structure	Parameters										
		λ_0_	α	Emax_TFR1_	TFR1_50_	γ1	Emax_CD44_	CD44_50_	γ2	Emax_FPN_	FPN_50_	γ3
BLCA	Gompertz	0.0073	-0.13	27.71	6.01	121.5	0.5	4.65	112.1	- 0.15	16.5	121.1
BRCA	Gompertz	0.0016	0.19	6.89	2.84	102.2	2.10	0.42	11.15	- 0.55	7.56	106.5
COAD	Gompertz	0.0076	0.032	10.31	9.85	137.5	0.25	0.12	3.20	- 0.27	11.05	124.5
HNSC	Weibull	0.020	-0.21	2.61	6.45	117.1	1.67	17.58	66.57	- 0.24	6.27	155.2
KICH	Weibull	0.0056	0.050	12.72	20.28	140.8	1.67	5.62	136.7	- 0.71	6.49	149.9
KIRC	Gompertz	0.0071	-0.049	14.31	1.75	180.6	1.96	18.19	15.36	- 0.92	25.52	1.64
KIRP	Weibull	0.043	0.21	2.14	10.29	18.09	0.86	1.35	134.1	- 0.58	9.93	149.8
LIHC	Weibull	0.19	-0.086	1.12	12.78	6.66	0.71	9.79	155.3	- 0.47	21.36	113.5
LUAD	Weibull	0.045	0.056	3.51	0.57	19.69	0.32	10.86	138.7	- 0.48	8.44	123.8
LUSC	Weibull	0.027	-0.08	5.42	5.22	12.91	0.80	148	125.7	- 0.22	57.33	127.6
PRAD	Weibull	0.00049	1.88	2.15E5	23.9	217.7	1.38	3.92	158.8	- 0.64	6.64	175.3
READ	Weibull	0.0004	0.57	54.42	8.39	138.9	4.5	23.22	161.2	- 0.62	95.64	121.5
STAD	Gompertz	0.045	0.066	4.44	0.91	17.61	1.87	10	100.6	- 0.58	8.21	130.2
THCA	Weibull	0.0014	0.39	19.44	4.07	217.4	0.84	6.07	15.88	- 0.70	16.29	70.91
UCEC	Weibull	0.0093	-0.0022	2.26	9.03	7.62	1.36	0.03	6.47	- 0.39	39.7	3.52

Note: Cancer types are labeled using the TCGA study abbreviations. $\:{\lambda\:}_{0}$ is the hazard scale factor, α is the hazard shape factor, $\:{{E}_{max}}_{TFR1}$, $\:{{E}_{max}}_{CD44}\:$, and $\:{{E}_{max}}_{FPN}$ are maximum effect parameters of TFR1, CD44, and FPN on OS, respectively. $\:{TFR1}_{50}$, $\:{CD44}_{50}$, and $\:{FPN}_{50}$ are half expression levels of TFR1, CD44, and FPN that are needed to reach the maximum effect on OS, respectively.The unit of $\:{TFR1}_{50}$, $\:{CD44}_{50}$, and $\:{FPN}_{50}$ is Transcripts Per Million (TPM). γ1, γ2, and γ3, are the slope-factor for the effect of TFR1, CD44, and FPN, respectively

These results are consistent with the findings that iron homeostasis alterations and cancer progression are interrelated, i.e. higher iron content is associated with shorter survival. These results indicate that the expression level of TFR1, CD44, and FPN has the clinical significance of prognostic biomarkers in response to cancer progression and predicted clinical outcomes.

The predictive performance was further validated by visual predictive check (VPCs). As shown in Figure S7, the VPCs stratified by cancer type showed good agreement between the observed data and model prediction, demonstrating acceptable predictive performance of the TTE models across cancers.

### CIRS stratifies the risk of long-term survival in cancers

To explore the potential of applying the alteration of iron homeostasis profiles to inform the prediction of long-term survival outcomes, we constructed CIRS based on the estimation results of the TTE models using the RNA-seq results of TFR1, CD44, and FPN (Table 1 and Table S5). The CIRS served as a feature extractor to translate the RNA-seq data into a list of 3 probabilities of the future incident risk, covering 15 types of cancers.

**Fig. 3:**
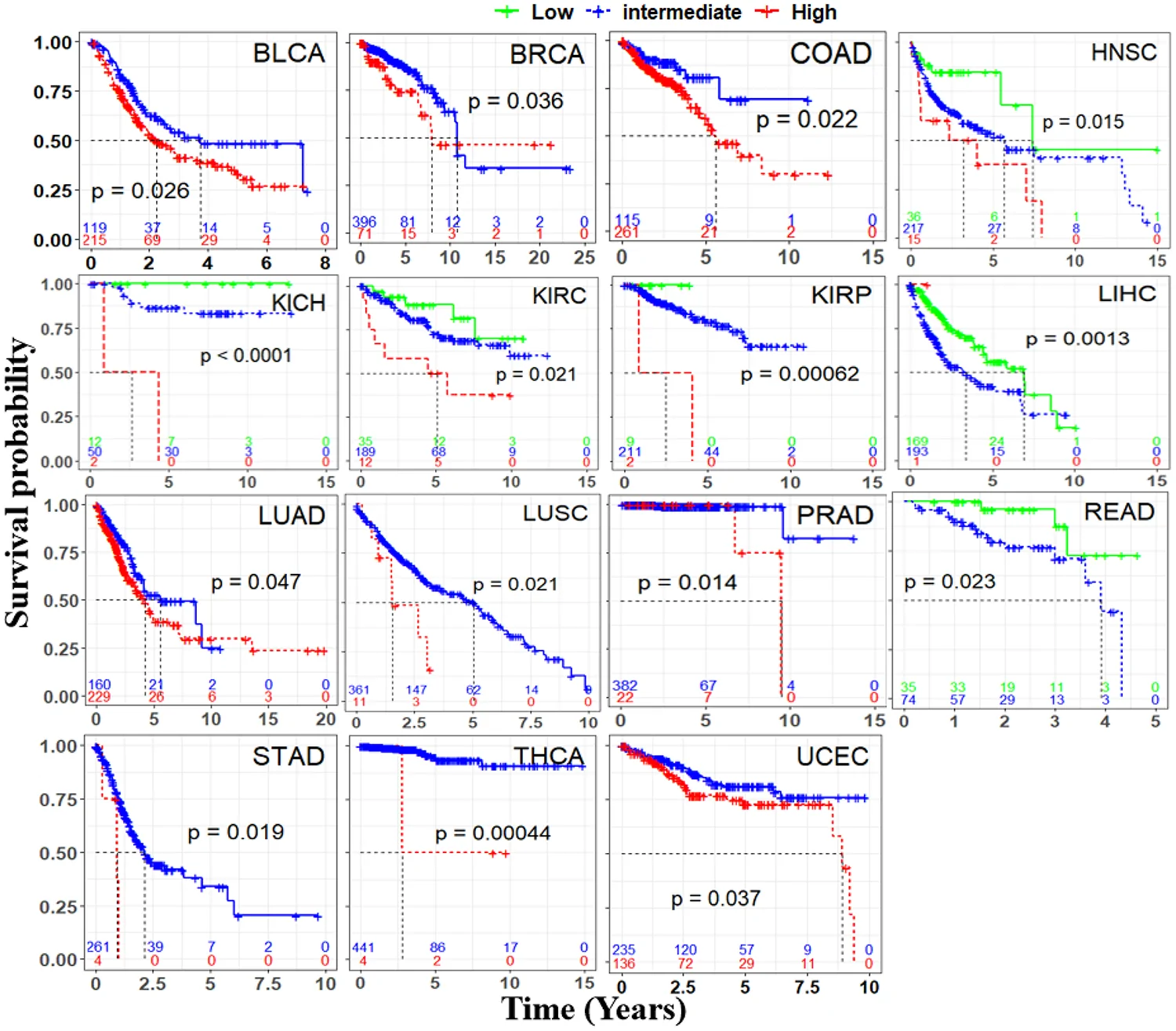
Cancer-specific iron risk score (CIRS) could be applied to stratify the survival probability in cancers. Kaplan-Meier survival plots of 15 cancer categories and all-cause mortality according to tertiles of CIRS during follow-up (green, bottom tertile; blue, median tertile; red, top tertile). Cancer types are labeled using the TCGA study abbreviations

As shown in Fig. 3, the Kaplan-Meier survival curves showed distinctive paths between the three risk groups stratified by CIRS across 15 cancers. The CIRS significantly stratified the cancer patients, and individuals in the high-risk group usually had notably worse outcomes and elevated risk of cancer progression and mortality. Of note, no low-risk group was identified in BLCA, BRCA, COAD, LUAD, LUSC, PRAD, STAD, THCA, and UCEC, while no high-risk group was identified in READ.

Overall, these results show that iron homeostasis features are typically associated with cancer progression, and CIRS has the potential to be useful measures to predict patients’ survival outcomes that can universally be applied to all cancers.

### Performance cross-validation of CIRS in external datasets

**Fig. 4:**
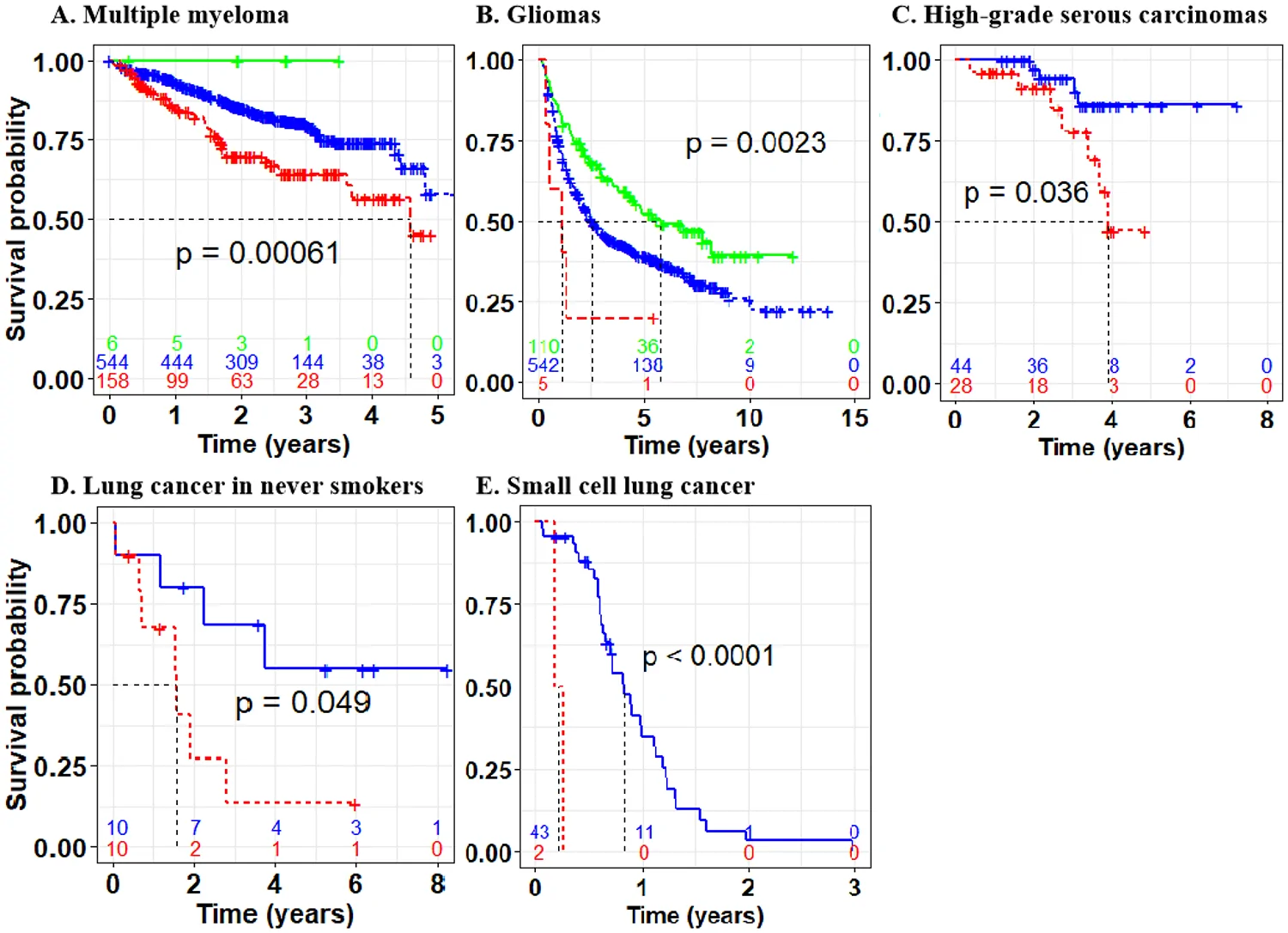
Evaluation of Cancer-specific iron risk score (CIRS) predictions across the five external validation datasets. CIRS effectively stratifies the survival probability in cancers. Kaplan-Meier survival plots of 5 cancer categories and all-cause mortality according to tertiles of CIRS during follow-up (green, bottom tertile; blue, median tertile; red, top tertile). **A**. Dataset of Multiple Myeloma from the CoMMpass study (NCT01454297). **B**. Dataset of Gliomas from the Chinese Glioma Genome Atlas (CGGA). **C**. Dataset of high-grade serous carcinomas (HGSCs). **D**. Dataset of lung cancer from the Sherlock-Lung study. **E**. Dataset of small cell lung cancer. The data accession numbers and references for these datasets and detailed clinical information are provided in Supplementary Table S6

We next conducted a comprehensive external and independent cross-validation of CIRS performance. We collected publicly available RNA-seq data and clinical information from primary tumor biopsies samples. The five datasets used for validation contained 1502 cancer patients spanning 4 cancer types (Table S6). Similar to the results obtained from TCGA, the cancer patients in the CIRS high-risk group usually have notably worse outcomes (Fig. 4). Overall, in this independent validation process, CIRS demonstrated the broad abilities to predict long-term survival of cancer patients.

### Iron homeostasis evolves during NACT and CIRS predicts NACT response in BRCA patients

**Fig. 5:**
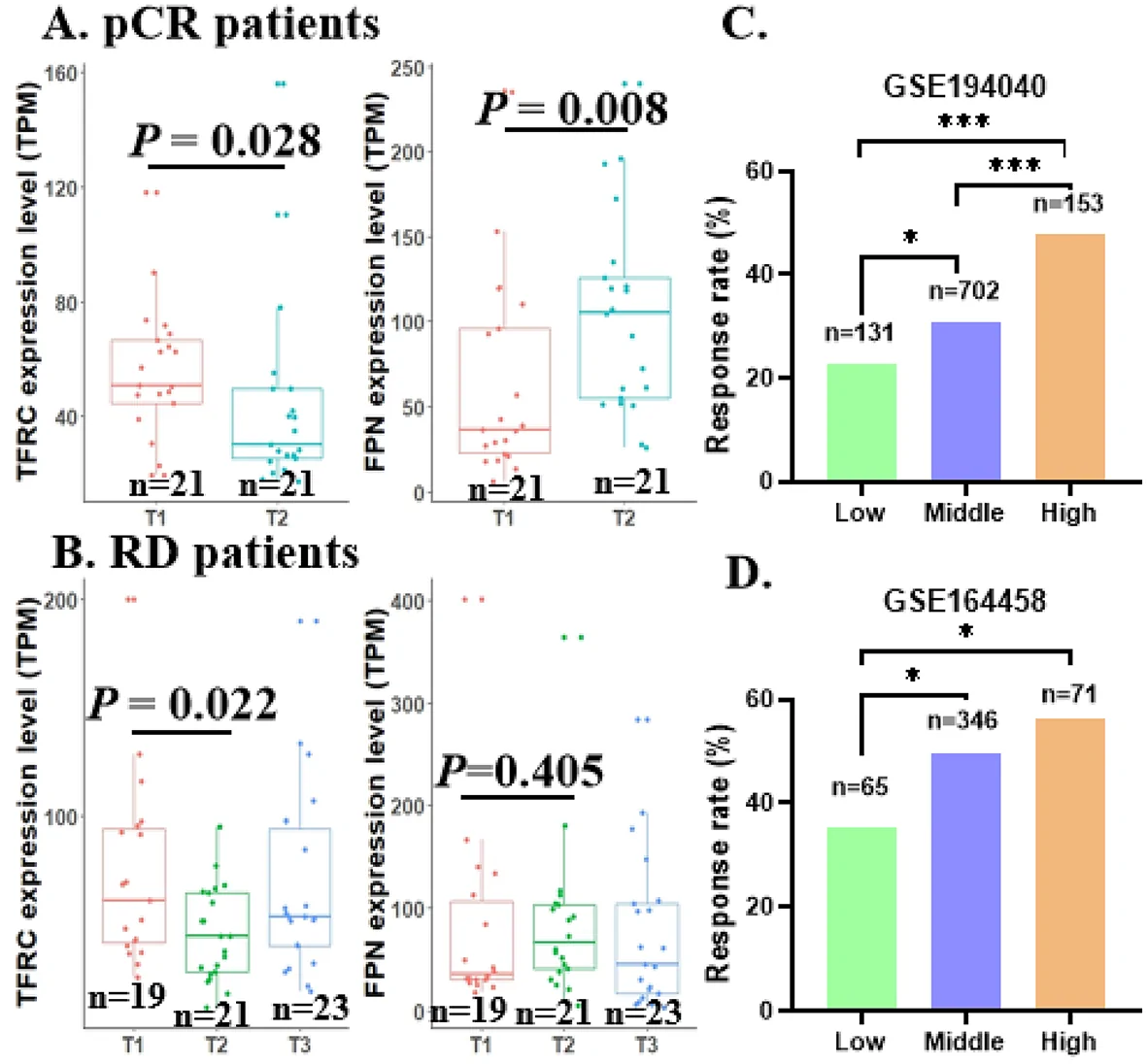
Iron homeostasis evolves during neoadjuvant chemotherapy (NACT), and the cancer-specific iron risk score (CIRS) could be applied to predict NACT response rate in breast cancer patients from two independent datasets (GSE194040 and GSE164458). **A**. Dynamic changes of expression of TFRC and FPN in patients achieved pathologic complete response (pCR). TPM = transcripts per million. **B**. Dynamic changes of expression of TFRC and FPN in patients harbored residual disease (RD) who were treated with standard NACT protocol. Tumor biopsies were collected for each patient at three time points—pre-treatment (T1), three weeks after the first cycle of NAC (T2) and at the time of surgery (T3). **C** and **D**. BRCA patients in the two datasets were classified as low, middle, and high risk based on CIRS as described in the methods. The response rate was calculated as the number of patients that achieved pathologic complete response (pCR) divided total number of patients in the subgroup. *p < 0.05. **p < 0.01, ***p < 0.001. Chi-Square tests

We next investigated whether CIRS could be used to predict NACT response in BRCA patients. Cancer cells require more iron for DNA and RNA replication, thus high CIRS should be more sensitive to NACT. We found that the expression of TFRC decreased significantly (*p* = 0.028) after NACT and the expression of FPN increased significantly (*p* = 0.008) accordingly in pCR patients (Fig. 5A), suggesting that NACT could inhibit BRCA to reduce the requirement of iron amount. However, in RD patients (Fig. 5B), the expression of FPN only showed a slightly increase trend compared to T1 (*p* = 0.405). Consistently, the expression of TFRC rebound and FPN further decreased at T3 in RD patients, which complies with the cancer recurrence. The evolutionary trajectories of iron homeostasis in BRCA progression were also observed in TCGA data. As shown in Figure S8, the TFRC exhibited an increased trend while the FPN decreased with the individual cancer stages advance (Figure S8A). Notably, this trend is consistent with the major subclass classification from luminal to HER2 positive to triple negative (Figure S8B) [32], the TP53 mutation status (Figure S8C) [33], and the nodal metastasis status (Figure S8D) [34]. Taken together, these analyses suggest that iron homeostasis evolves with BRCA progression, supporting tumor proliferation, aggressiveness, and metastatic potential.

We further demonstrated that the CIRS is capable of predicting the NACT response in BRCA patients. In the dataset from GSE194040 (Fig. 5C), the NACT response rates were 22.9%, 30.77%, and 47.71% in the low, middle, and high risk groups, respectively defined by CIRS (Chi-Squared test, *p* < 0.001). This result was validated using an independent dataset GSE164458 (Fig. 5D), the NACT response rate increased from 35.38% in the low risk group to 49.71% in the middle risk group and 56.34% in the high risk group (Chi-Squared test, *p* < 0.05). This observation confirms the association between CIRS and NACT response rates, as high CIRS have higher NACT response rates compared with middle and low CIRS.

## Discussion

Population-based studies collectively support a model in which increased intracellular iron retention worsens disease outcomes in cancer [35]. Substantive evidence implicates changes in the uptake and efflux of iron as crucial features of cancer to meet the increased demands for iron to support rapid proliferation and evasion [7, 29, 36]. Although some empirical studies facilitated the understanding of the complex relationship between iron and cancer, the precise contributions of the expression of iron transport genes on cancer progression are not yet fully understood. A more comprehensive picture of exactly how iron homeostasis is altered in cancers in a quantitative manner would be of substantial benefit for identifying key points of control that can be therapeutically manipulated [37, 38].

Leveraging systematic information from RNA-seq profiling in CCLE and TCGA, we discovered that at least one iron uptake gene, either TFR1 or CD44 was overexpressed, or the only known iron efflux gene FPN was decreased, or both, across cancer types. To prioritize iron-related genes from a relatively large candidate set, we implemented a machine-learning approach using XGBoost. This approach was selected because it can evaluate multiple correlated gene-expression features simultaneously and identify the variables with the greatest prognostic contribution in a data-driven manner, rather than relying only on predefined assumptions or single-gene comparisons [39]. In the present study, the main advantage of this approach was that it enabled robust feature prioritization across heterogeneous cancer transcriptomic datasets and helped focus the downstream time-to-event modeling on the most informative iron-handling genes. Notably, we also found significant correlations between the alteration of these three genes and patient survival. Moreover, we successfully developed a parametric TTE model combining individual patient baseline TFR1, CD44, and FPN RNA levels to quantify the contribution of these three genes to patients’ OS across 15 cancers. Using the model estimation results, we developed and validated a CIRS that markedly stratified the risk of cancer patients and predicted NACT response in BRCA. Importantly, high CIRS was associated with better NACT response in breast cancer. This association can be explained by the critical role of iron in supporting cancer cell proliferation and survival. Cancer cells require elevated levels of iron to sustain DNA and RNA synthesis, which are essential for their rapid growth and replication. High CIRS reflects increased intracellular iron retention, which supports tumor aggressiveness but also creates a therapeutic vulnerability. Specifically, excess iron exacerbates oxidative stress via the Fenton reaction, leading to the generation of reactive oxygen species (ROS) and increased DNA damage. This heightened baseline oxidative stress makes cancer cells with high CIRS more sensitive to DNA-damaging chemotherapeutic agents, as these drugs further amplify oxidative damage and disrupt DNA integrity. Therefore, high CIRS identifies a tumor phenotype that is both aggressive and highly susceptible to cytotoxic NACT. These findings are consistent with the inverse relation showing that increased intracellular iron retention is associated with worse OS in various cancers and with greater sensitivity to the DNA-damaging chemotherapeutic drugs in BRCA.

Distinct from prior and recent works, our study is unique in providing intriguing insights into quantifying the contribution of iron homeostasis alteration by modeling cancer progression and long-term survival outcomes [3, 4, 40, 41]. By simplifying the iron homeostasis alteration into the expression level changes of three iron transport genes that can be measured quantitatively, the model subtly connects the RNA level of the genes with survival outcomes and NACT response. This enabled the characterization of the impact of changing iron transport genes on survival and treatment outcomes in cancer. This further facilitated simulation-based exploration of long-term survival with different iron transport gene expression levels.

The alteration of iron homeostasis and the addiction of cancer cells to iron has led to three broad strategies in cancer therapy, including the use of iron chelators to deplete cancer cells of iron, ferroptosis inducers towards selective cytotoxicity to cancer cells, and iron transporters as the target to deliver cytotoxic payloads preferentially to tumor cells [7, 42]. Although the promising prospects for the potential of iron modulation as a strategy to treat advanced cancers, the results are not always consistent. For example, iron chelators (such as Deferoxamine and Deferasirox), either alone or in combination with chemotherapy, had been tested to treat cancers by depriving cancer cells of the iron that they need for their rapid growth [38]. Despite some chelators performing well against several cancers, some chelators fail to achieve the desired efficacy [6]. A phase II trial demonstrated that Deferoxamine decreased bone marrow infiltration in 7 of 9 patients with neuroblastoma [43]. However, another phase II trial suggested that Deferoxamine has no effect on metastatic prostate cancer as 13 of 14 patients had disease progression [44]. In addition, in a clinical trial of Deferoxamine for advanced hepatocellular carcinoma (*n* = 10), 2 patients had a partial response, 3 had stable disease, and 5 had progressive disease [45]. This iron-target treatment heterogeneity is a remaining question that needs to be addressed.

Currently, the availability of high-throughput gene sequencing technology led to a deeper understanding of the significant heterogeneity across cancers and accelerated the development of precision oncology [46, 47]. The ability to predict which treatment is likely to be effective based on individual patients’ distinct iron homeostasis profiles can be transformative for cancer therapy [42]. In this study, we demonstrated how RNA-seq data of the expression level of iron transport genes might inform precision oncology when treating patients with iron chelators or ferroptosis inducers.

To our knowledge, this is the first study to investigate the interconnection between iron homeostasis and patients’ survival and treatment outcomes across cancers from a quantitative perspective using a TTE modeling approach. A single gene is unlikely to fully control iron homeostasis, leading to bias in predicting the risk of cancer patients accurately. By mapping iron transportation and progression dynamics in cancer patients, our finding relating high expression of TFR1, CD44 and low expression of FPN with worse OS across cancers is consistent with previous works [48]. RNA-seq results reflecting human genetic information and health status are central to clinical decision-making. Integrating the genomic data of RNA-seq techniques, the CIRS we constructed, based on both uptake and efflux, stratified risk trajectories well across 15 cancers studied. The patient who is deemed as high risk by CIRS holds the greater potential to benefit from iron perturbation therapy. This mathematical model-based integration of knowledge from diverse sources represents a more efficient use of genomic data and hence more apt to maximize efficacy and/or reduce treatment-related heterogeneity, and is in line with the principles of model-informed precision dosing for treatment optimization [49, 50]. We developed the CIRS based on the cancer patients’ data, which is essential for the early identification of individuals specifically in need of iron-targeting therapeutic interventions. The three genes we identified in this study were associated with different cancer types in a highly concordant manner, interventions on identified shared pathways are likely to deliver benefits in a consistent manner without increasing additional risk. In this scenario, TFR1, CD44, and FPN may serve as attractive targets for cancer intervention given their robust predictive power for various cancer types.

Our analyses have several advantages. Our study defined the EC_50_ cutoff value of gene expression level to effectively distinguish cancer patients and predict survival benefits, the RNA-seq-based simple classifier CIRS not only exhibited favorable performance, but also is much simpler and easier for implementation than the existing method which usually requires a large amount of data and advanced computational method [51]. Second, this study also generates new hypotheses that warrant further investigation. For example, iron-target treatment should differ considerably due to the significant heterogeneity in the expression level of iron transport genes, with the increase of iron uptake genes and the decrease of iron efflux gene negatively correlating with cancer progression and patient survival outcomes. The association between the dynamic changes of the expression level of these genes during cancer progression and the iron-target treatment efficacy have been relatively underexplored in the literature and could be important directions for future work.

This study has one limitation that merit further discussion and exploration in future research. There are five genes that partially participate in iron uptake were not included in the TTE models during initial screening and thus excluded for developing CIRS. Yet, the purpose of this study was to assess the feasibility of using major iron uptake and efflux genes that can universally be applied to all cancers for clinical use, not to employ all genes. We provide preliminary evidence for the predictive value of these genes in multiple cancers. However, we cannot rule out the possibility that the predictive ability of other unidentified genes that may participate in iron transport may be superior for specific cancer types, and future relevant studies are needed to verify our results.

## Conclusions

In conclusion, we quantified the impact of iron homeostasis alteration on patients’ survival across diverse cancers and NACT response in BRCA by modeling the expression level of iron uptake genes TFR1 and CD44, and efflux gene FPN. This study provided useful insights to identify cancer patient subgroups at higher risk based on predefined CIRS using RNA-seq data, and guide optimal clinical interventions regarding the significant predictors, which would constitute a novel strategy for cancer diagnosis, staging, and lead to potential therapeutic strategies to target these tumor-specific events.

## Supplementary Information

Below is the link to the electronic supplementary material.

Supplementary Material 1

## Data Availability

The data that support the findings of this study are available in Cancer Cell Line Encyclopedia at https://sites.broadinstitute.org/ccle; Expression Atlas at https://www.ebi.ac.uk/gxa/home; The Cancer Genome Atlas (TCGA) at https://portal.gdc.cancer.gov/; Clinical Proteomic Tumor Analysis Consortium (CPTAC) at https://proteomics.cancer.gov/programs/cptac; Gene Expression Omnibus (GEO) at https://www.ncbi.nlm.nih.gov/geo/; and Human Protein Atlas (HPA) at https://www.proteinatlas.org/. The code used to analyze data in this paper are available at the Supplementary Information. The code and data are also available from the authors upon reasonable request.

## Abbreviations

BRCA: Breast invasive carcinoma
BLCA: Bladder urothelial carcinoma
CCLE: Cancer Cell Line Encyclopedia
CD44: Cluster determinant 44
CIRS: Cancer-specific iron risk score
COAD: Colon adenocarcinoma
CPTAC: Clinical Proteomic Tumor Analysis Consortium
DMT1: Divalent metal transporter 1
ESCA: Esophageal carcinoma
FPN: Ferroportin
GDC: Genomic Data Commons
HCP1: Heme carrier protein
HNSC: Head and neck squamous cell carcinoma
HPA: Human Protein Atlas
KICH: Kidney chromophobe
KIRC: Kidney renal clear cell carcinoma
KIRP: Kidney renal papillary cell carcinoma
LCN2: Lipocalin-2
LIP: Labile iron pool
LIHC: Liver hepatocellular carcinoma
LUAD: Lung adenocarcinoma
LUSC: Lung squamous cell carcinoma
NTBI: Non–transferrin-bound iron
OS: Overall survival
PRAD: Prostate adenocarcinoma
READ: Rectum adenocarcinoma
STAD: Stomach adenocarcinoma
TCGA: The Cancer Genome Atlas
TF: Transferrin
TFR1: Transferrin receptor 1
THCA: Thyroid carcinoma
TTE: Time-to-event
UCEC: Uterine corpus endometrial carcinoma
VPCs: Visual predictive checks
