# Supplementary material for: Iron homeostasis landscape informs patient survival and neoadjuvant chemotherapy response in cancer
Source: BioData Min. 2026 Jul 24;19:82. doi: 10.1186/s13040-026-00589-3 (PMC13685333; doi:10.1186/s13040-026-00589-3)
Supplement: Supplementary file 1 [file 13040_2026_589_MOESM1_ESM.docx]

Supplemental information

**Iron Homeostasis Landscape Informs Patient Survival and Neoadjuvant Chemotherapy Response in Cancer**

*Xiaoqing Fan,* *Kangna Cao,* *Xu Steven Xu, and Xiaoyu Yan**

**Table S1.** Summary of iron transport‐related proteins.

| **Function** | **Name** | **Gene symbol** | **Ensembl** | **Description** |
| --- | --- | --- | --- | --- |
| Uptake | CD44 | CD44 | ENSG00000026508 | Hyaluronate/cluster determinant 44 |
|  | DMT1 | SLC11A2 | ENSG00000110911 | Divalent metal transporter 1 |
|  | HCP1 | SLC46A1 | ENSG00000076351 | Heme carrier protein |
|  | LCN2 | SLC22A17 | ENSG00000092096 | Solute carrier family 22 member 17, lipocalin-2 |
|  | TFR1 | TFRC | ENSG00000072274 | Transferrin receptor 1 |
|  | ZIP14 | SLC39A14 | ENSG00000104635 | Solute carrier family 39 member 14, transmembrane ion channel |
|  | ZIP8 | SLC39A8 | ENSG00000138821 | Solute carrier family 39 member 8, transmembrane ion channel |
| Efflux | FPN | SLC40A1 | ENSG00000138449 | Solute carrier family 40 member 1, ferroportin |

| **Tissue types** | **Sample size** | |
| --- | --- | --- |
|  | **Cancer cell lines** | **Normal tissues** |
| Adrenal | 1 | 5 |
| Bladder | 13 | 5 |
| Blood | 16 | 2 |
| Bone | 12 | 2 |
| Brain | 42 | 21 |
| Breast | 59 | 2 |
| Colon | 11 | 7 |
| Endometrium | 6 | 2 |
| Esophageal | 10 | 2 |
| Head and neck | 9 | 1 |
| Kidney | 15 | 7 |
| Liver | 15 | 5 |
| Lung | 149 | 5 |
| Lymph node | 4 | 3 |
| Ovary | 40 | 5 |
| Pancreas | 13 | 2 |
| Prostate | 7 | 4 |
| Rectum | 2 | 1 |
| Skeletal muscle | 1 | 2 |
| Skin | 26 | 5 |
| Stomach | 14 | 3 |
| Testis | 1 | 7 |
| Thyroid | 3 | 4 |
| Uterus | 9 | 3 |

**Table S2.** Summary of Cancer Cell Line Encyclopedia (CCLE) and human normal tissue samples data downloaded from Expression Atlas.

**Table S3.** Summary of the databases used in this study.

| **Database** | | **Description** | | **Link** | |
| --- | --- | --- | --- | --- | --- |
| **Cancer Cell Line Encyclopedia (CCLE)** | | A compilation of gene expression, chromosomal copy number and massively parallel sequencing data from human cancer cell lines. | | https://sites.broadinstitute.org/ccle | |
| **Expression Atlas (EA)** | | An open science resource that provides gene expression results on more than 3,000 experiments from 40 different organisms. | | https://www.ebi.ac.uk/gxa/home | |
| **Gene Expression Omnibus (GEO)** | | A public functional genomics data repository | | https://www.ncbi.nlm.nih.gov/geo | |
| **UALCAN** | **The Cancer Genome Atlas (TCGA)** | Contains demographic information, protein-coding gene expression data, and survival of cancer patients from primary cancer and matched normal samples spanning 33 cancer types. | A comprehensive database that integrates publicly available cancer OMICS data (including TCGA, CPTAC, and HPA) | https://portal.gdc.cancer.gov | https://ualcan.path.uab.edu/index.html |
|  | **Clinical Proteomic Tumor Analysis Consortium (CPTAC)** | Contains proteogenomic data. |  | https://proteomics.cancer.gov/programs/cptac |  |
|  | **Human Protein Atlas (HPA)** | Contains demographic information, survival data, and the expression profiles in human tissues of genes both on the mRNA and protein level of cancer patients. |  | https://www.proteinatlas.org |  |

**Table S4.** Demographic information of cancer patients for TTE model development in the present study from TCGA

| **Cancer types** | **Characteristics** | | | | | | |
| --- | --- | --- | --- | --- | --- | --- | --- |
|  | **Sample size** | | **Age, years (mean±SD)** | **Ethnicity (white)** | **TFR1 (median, range)** | **CD44 (median, range)** | **FPN (median, range)** |
|  | **Male** | **Female** |  |  |  |  |  |
| **BLCA** | 265 | 85 | 68.15±10.73 | 282 (80.57%) | 21.3 (2.5, 330.6) | 27.2 (0.7, 560.1) | 8.35 (0.5, 349.2) |
| **BRCA** | 7 | 460 | 60.46±13.81 | 321 (68.74%) | 14.8 (1.9, 118.6) | 41.7 (2.4, 681.7) | 37.9 (0.1, 783.3) |
| **COAD** | 202 | 174 | 66.34±13.21 | 178 (47.34%) | 51.85 (3.3, 171.5) | 35.8 (3.3, 108.6) | 49.05 (3.2, 299.2) |
| **HNSC** | 184 | 84 | 63.64±11.98 | 240 (89.55%) | 22.1 (2.8, 324.6) | 97.3 (15.9, 507.2) | 6.4 (0.4, 96.7) |
| **KICH** | 38 | 26 | 51.58±13.94 | 58 (90.63%) | 16.8 (1.2, 151.9) | 11.8 (0.8, 64.1) | 19.4 (3, 112.4) |
| **KIRC** | 146 | 90 | 60.84±11.41 | 194 (82.20%) | 9.15 (1.4, 50.1) | 12.7 (1, 358.2) | 45.55 (1.6, 175.1) |
| **KIRP** | 167 | 55 | 62.01±11.72 | 160 (70.07%) | 3.75 (0.6, 169.6) | 8.65 (0.2, 198.9) | 38.05 (1.6, 112.8) |
| **LIHC** | 246 | 117 | 59.54±13.31 | 181 (49.86%) | 42.4 (5, 519) | 9 (1, 73.2) | 2.6 (0.1, 96.4) |
| **LUAD** | 177 | 212 | 65.65±9.96 | 310 (79.69%) | 14.8 (1.7, 154.9) | 31.4 (2.5, 166.1) | 37.2 (4, 308.5) |
| **LUSC** | 281 | 105 | 67.90±8.64 | 288 (74.61%) | 67.65 (3.6, 1297.9) | 67.55 (1.4, 349.5) | 20.45 (0.8, 260.5) |
| **PRAD** | 404 | 0 | 61.16±6.95 | 337 (83.42%) | 3.7 (0.5, 24.6) | 19 (0.4, 87) | 14.8 (2, 57.3) |
| **READ** | 63 | 48 | 64.21±11.63 | 61 (54.95%) | 42.5 (7.4, 160.3) | 31.5 (11.3, 80.8) | 48.8 (6, 344.3) |
| **STAD** | 172 | 93 | 66.17±10.62 | 193 (72.83%) | 22.5 (2.7, 150) | 33.6 (2.8, 1153.6) | 44.1 (1.8, 229.8) |
| **THCA** | 124 | 321 | 47.55±15.91 | 290 (65.17%) | 5 (0.7, 25.8) | 52.4 (6.5, 167.6) | 39.2 (7.2, 148.5) |
| **UCEC** | 0 | 377 | 64.67±11.49 | 271 (71.88%) | 19.1 (1.5, 273.8) | 17.8 (1.2, 139.7) | 43.9 (1.1, 670) |

**Notes:** Cancer types are labeled using the TCGA study abbreviations. BLCA: Bladder urothelial carcinoma; BRCA : Breast invasive carcinoma; COAD : Colon adenocarcinoma; ESCA : Esophageal carcinoma; HNSC : Head and Neck squamous cell carcinoma; KICH : Kidney chromophobe; KIRC : Kidney renal clear cell carcinoma; KIRP : Kidney renal papillary cell carcinoma; LIHC : Liver hepatocellular carcinoma; LUAD : Lung adenocarcinoma; LUSC : Lung squamous cell carcinoma; PRAD : Prostate adenocarcinoma; READ : Rectum adenocarcinoma; STAD: Stomach adenocarcinomna; THCA: Thyroid carcinoma; UCEC : Uterine corpus endometrial carcinoma. The unit of TFR1, CD44, and FPN expression level is Transcripts Per Million (TPM).

**Table S5.** Cancer-specific iron risk score (CIRS) development based on the TTE modeling estimation results.

| **Proteins** | **Risk** | | |
| --- | --- | --- | --- |
|  | **High** | **Low** | **Intermediate** |
| **TFR1** | > TFR1_50_ | < TFR1_50_ | The patient outside the high and low-risk group |
| **CD44** | > CD44_50_ | < CD44_50_ |  |
| **FPN** | < FPN _50_ | >FPN_50_ |  |

**Table S6.** Summary of the five external datasets used in this study for cross-validation of CIRS.

| **Datasets** | **Sample size** | **Brief information** | **Reference** |
| --- | --- | --- | --- |
| 1 | 708 | Tumor samples from patients with multiple myeloma are characterized at diagnosis and progression. | Nat Genet. 2024; 56(9):1878-1889.  **doi**: 10.1038/s41588-024-01853-0.  **PMID**: 39160255 |
| 2 | 657 | Chinese Glioma Genome Atlas (CGGA), a user-friendly data portal, including glioma samples from Chinese cohort. | Genomics Proteomics Bioinformatics. 2021; 19(1):1-12.  **doi**: 10.1016/j.gpb.2020.10.005.  **PMID**: 33662628 |
| 3 | 72 | A multi-center integrated genomic analysis of high-grade serous carcinomas. | Nat Commun. 2017; 8(1):990.  **doi**: 10.1038/s41467-017-01217-9.  **PMID**: 29042553 |
| 4 | 20 | RNA-sequencing data and patient-matched clinical information were obtained from treatment-naïve never smoker patients with lung cancer. | Nat Genet. 2021 Sep; 53(9):1348-1359.  **doi**: 10.1038/s41588-021-00920-0.  **PMID**: 34493867 |
| 5 | 45 | Tumours and clinical characteristics from patients with small cell lung cancer (SCLC). | Nature. 2024 Mar; 627(8005):880-889. **doi**: 10.1038/s41586-024-07177-7.  **PMID**: 38480884 |

**Table S7.** Detailed information of the 2 independent datasets used for CIRS to predict neoadjuvant chemotherapy response rate.

| **GEO**  **series** | **Subtype** | **Treatment** | **Sample size** | | **Ref** |
| --- | --- | --- | --- | --- | --- |
|  |  |  | **Res** | **Non-res** |  |
| GSE194040 | ER, TNBC | Pac, Dox, Cyc, Gane | 26 | 67 | [42] |
|  | ER, TNBC | Pac, Dox, Cyc, Tre | 33 | 82 |  |
|  | ER, TNBC | Pac, Dox, Cyc | 31 | 148 |  |
|  | ER, TNBC | Pac, Dox, Cyc, Gani | 24 | 81 |  |
|  | ER, TNBC | Pac, Dox, Cyc, MK-2206 | 18 | 42 |  |
|  | ER, HER2, TNBC | Pac, Dox, Cyc, Ner | 42 | 72 |  |
|  | ER, TNBC | Pac, Dox, Cyc, Pem | 31 | 38 |  |
|  | HER2 | Pac, Dox, Cyc, Per, Tra | 26 | 18 |  |
|  | HER2 | Pac, Dox, Cyc, Tra, Emt, Per | 30 | 22 |  |
| GSE164458 | TNBC | Pac, Dox, Cyc, Car | 69 | 53 | [41] |
|  | TNBC | Pac, Dox, Cyc, Car, Vel | 127 | 110 |  |

**Notes**: Car = carboplatin; Cyc = cyclophosphamide; Dox = doxorubicin; Emt = emtansine; ER = estrogen receptor; Gane = ganetespib; Gani = ganitumab; HER2 = human epidermal growth factor receptor-2; Ner = neratinib; NON-res = Non-responder; Pac = paclitaxel; Per = pertuzumab; Pem = pembrolizumab; Ref = reference; Res = responder; TNBC = triple-negative; Tra = trastuzumab; Tre = trebananib; Vel = veliparib.

**Figure S1**. Cancer cell lines demonstrate higher expression of transferrin receptor 1 (TFR1) or CD44 for iron uptake, or lower expression of Ferroportin (FPN) for iron export, or both, that favor iron accumulation to promote oncogenesis and cell proliferation. A. The expression level of TFR1 (upper panel), CD44 (middle panel), and SLC40A1 (bottom panel) in different types of tumors compared to the corresponding normal tissues. B. Heatmap of the expression level of TFR1, CD44, and FPN in different types of tumors compared to the corresponding normal tissues. Data presented as Log10(fold change) of tumors vs normal tissues; color was scaled relative to the decrease (blue) and increase (red) values of each cancer. FPKM = Fragments Per Kilobase of exon per Million reads. **p* < 0.05. ***p* < 0.01, ****p* < 0.001.*****p* < 0.0001. Multiple unpaired t-tests. The sample size within each tissue is listed in Table S2.

**Figure S2.** The expression level of DMT1 (A), HCP1 (B), LCN2 (C), ZIP8 (D), and ZIP14 (E) in different types of tumors cells compared to the corresponding normal tissues. FPKM = Fragments Per Kilobase of exon per Million reads. **p* < 0.05. ***p* < 0.01, ****p* < 0.001.*****p* < 0.0001. ns = no significant difference. Multiple unpaired t test.

**B**

**E**

**D**

**C**

**A**

**Figure S3.** Schematic overview of the key players in the cellular iron homeostasis handling machinery. Small red circles represent iron. Cellular iron homeostasis is mainly maintained by iron uptake and efflux. Classically, iron is absorbed by TF-TFR1 and CD44-mediated endocytosis. Following endocytosis, the iron in the labile iron pool will participate in the synthesis of iron-sulfur clusters (ISCs) and heme, which are critical cofactors of numerous proteins in the electron transport chain (ETC) and tricarboxylic acid (TCA) cycle in the mitochondrial matrix. Excess iron is exported outside the cell via ferroportin (FPN). Abbreviations: LIP, labile iron pool; TF, transferrin; TFR1, transferrin receptor protein 1.

**Figure S4.** Protein expression of TFRC (upper panel) and CD44 (lower panel) in normal and paired tumors based on cancer types from the CPTAC database. *p* value in black color means the expression level trend of the protein is different from our hypothesis, while the *p* value in red color means the expression level trend of the protein is consistent with our hypothesis. *P* value in blue color means no significant difference.

**Figure S5.** Phospho protein expression of TFRC (upper panel) and CD44 (lower panel) in normal and paired tumors based on cancer types from the CPTAC database. *p* value in black color means the expression level trend of the protein is different from our hypothesis, while the *p* value in red color means the expression level trend of the protein is consistent with our hypothesis. *P* value in blue color means no significant difference.

**Figure S6**. Ranking of importance of iron transport protein features on cancer patients’ overall survival. The bar chart indicates the importance of the sorted iron transport proteins based on their contributions to the prediction of OS (as judged by the information gain). Cancer types are labeled using the TCGA study abbreviations.

**Figure S7**. Visual predictive check for the time-to-event models of survival in different types of cancers. The solid line in each panel represents the observed Kaplan–Meier curve. The shaded area shows 95% confidence interval of the model-predicted Kaplan–Meier curve based on 1000-time simulations. Cancer types are labeled using the TCGA study abbreviations. BLCA: Bladder urothelial carcinoma; BRCA : Breast invasive carcinoma; COAD : Colon adenocarcinoma; ESCA : Esophageal carcinoma; HNSC : Head and Neck squamous cell carcinoma; KICH : Kidney chromophobe; KIRC : Kidney renal clear cell carcinoma; KIRP : Kidney renal papillary cell carcinoma; LIHC : Liver hepatocellular carcinoma; LUAD : Lung adenocarcinoma; LUSC : Lung squamous cell carcinoma; PRAD : Prostate adenocarcinoma; READ : Rectum adenocarcinoma; STAD: Stomach adenocarcinomna; THCA: Thyroid carcinoma; UCEC : Uterine corpus endometrial carcinoma.

**Figure S8.** Evolutionary trajectories of iron homeostasis. A. Expression of TFRC and FPN in normal subjects, stage 1, stage 2, stage 3, and stage 4 patients. B. Expression of TFRC and FPN in normal subjects, luminal patients, HER2 positive patients, and triple negative (TNBC) patients. C. Expression of TFRC and FPN in normal subjects, TP53 non-mutant patients, and TP53-mutant patients. D. Expression of TFRC and FPN in normal subjects, No regional lymph node metastasis patients, N1 metastases in 1 to 3 axillary lymph nodes patients, N2 metastases in 4 to 9 axillary lymph nodes patients, and N3 metastases in 10 or more axillary lymph nodes patients.

**A. Individual cancer stages**

**B. Major subclasses**

**C. TP53 Mutation status mutation status**

**D. Nodal metastasis status**

**Supporting information**

**CODE EXAMPLE**:

$PROBLEM SURVIVAL ANALYSIS MODEL

$INPUT	ID DV CMT TIME TTE MDV SEX AGE FPKMTFRC FPKMCD44 FPKMSLC

$DATA UCEC_SURV_FINAL.csv IGNORE=@

$SUBROUTINES ADVAN6 TOL=6

$MODEL

NCOMPARTMENTS=1

COMP=(CPT1)	; TTE model

$PK

TVBASE = THETA(1)

BASE	= TVBASE*EXP(ETA(1))

LAMB	= BASE

BETA	= THETA(2)

DEL = 1E-8

SMAX1	= THETA(3)

SC501	= THETA(4)

HILL1	= THETA(5)

EFF1	= 1+SMAX1*FPKMTFRC**HILL1/(FPKMTFRC**HILL1+SC501**HILL1)

SMAX2	= THETA(6)

SC502	= THETA(7)

HILL2	= THETA(8)

EFF2	= 1-SMAX2*FPKMSLC**HILL2/(FPKMSLC**HILL2+SC502**HILL2)

SMAX3	= THETA(9)

SC503	= THETA(10)

HILL3	= THETA(11)

EFF3	= 1+SMAX3*FPKMCD44**HILL3/(FPKMCD44**HILL3+SC503**HILL3)

$DES

DADT(1)= LAMB*EXP(BETA*LOG((T + DEL)))*EFF1*EFF2*EFF3	; hazard

$ERROR

CHZ = A(1)

SUR = EXP(-CHZ)

HAZ = LAMB*EXP(BETA*LOG((TIME + DEL)))*EFF1*EFF2*EFF3

IF (DV.EQ.0.AND.TTE.EQ.1) THEN

PR	= SUR

ENDIF

IF (DV.EQ.1.AND.TTE.EQ.1) THEN

PR	= SUR*HAZ

ENDIF

Y=PR

$THETA

(0, 0.01) ; Rate factor

0.01 ; Shape factor

(0,1)	;THETA3

(0,1)	;THETA4

(0,1)	;THETA5

(0,0.1,1)	;THETA6

(0,1)	;THETA7

(0,1)	;THETA8

(0,1)	;THETA9

(0,1)	;THETA10

(0,1)	;THETA11

$OMEGA

0 FIX	; ETA1 ON SD

$ESTIMATION MAXEVAL=9999 POSTHOC METHOD=COND PRINT=1 LAPLACIAN LIKE NUMERICAL SLOW NOABORT SIGL=9 NSIG=1

$COVARIANCE MATRIX=S UNCONDITIONAL SLOW SIGL=12 PRINT=E

$TABLE ID TIME DV CMT MDV TTE SUR HAZ CHZ AGE SEX FPKMSLC FPKMTFRC NOPRINT ONEHEADER FILE=mytab2 FORMAT=s1PE17.9
